# 5-O-Methylvisammioside alleviates depression-like behaviors by inhibiting nuclear factor kappa B pathway activation via targeting SRC

**DOI:** 10.4103/NRR.NRR-D-24-00714

**Published:** 2025-04-29

**Authors:** Wenqian Zhu, Bingjin Li, Ranji Cui

**Affiliations:** Jilin Provincial Key Laboratory of Molecular and Chemical Genetic, Second Hospital of Jilin University, Changchun, Jilin Province, China

**Keywords:** 5-O-methylvisammioside, depression-like behavior, hippocampus, major depressive disorder, microglia, network pharmacology, neuroinflammation, nuclear factor kappa B signaling pathway, PP2, Src

## Abstract

Preliminary studies on emerging herbal ingredients have highlighted alternative pathways that inflammation and modulate perturbed immunity as valuable strategies for treating depression. Previous studies have shown that 5-O-methylvisammioside, a bioactive compound derived from Saposhnikoviae Radix, possesses excellent anti-inflammatory and antioxidant biological functions, exhibits a neuroprotective effect. The purpose of this study was to explore the targets and signaling pathways of 5-O-methylvisammioside in the potential treatment of major depressive disorder using a combination of network pharmacology analysis and biological experiments. The network pharmacological analysis results indicated that the proto-oncogene tyrosine-protein kinase Src and the nuclear factor kappa B signaling pathway were highly correlated with the treatment of major depressive disorder with 5-O-methylvisammioside. Further experiments indicated that 5-O-methylvisammioside significantly improved lipopolysaccharide-induced depression-like behaviors in mice, ameliorated microglial polarization in the hippocampal CA1 and CA3 regions, and inhibited Src phosphorylation and nuclear factor kappa B pathway activation. The effects of 5-O-methylvisammioside were similar to those of the Src inhibitor PP2. When 5-O-methylvisammioside was administered with PP2, no effects were observed on lipopolysaccharide-induced depression-like behaviors in mice, nuclear factor kappa B pathway proteins, and microglial polarization. These findings indicate that 5-O-methylvisammioside may exert its antidepressant potential by inhibiting Src-mediated activation of the nuclear factor kappa B signaling pathway. Therefore, 5-O-methylvisammioside might serve as a promising Chinese herbal medicine for the prevention and treatment of depression.

## Graphical abstract

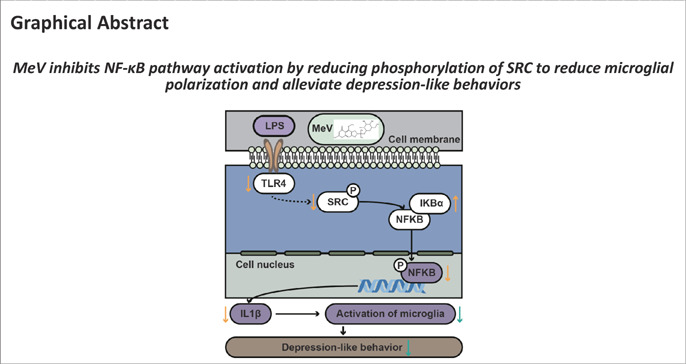

## Introduction

Major depressive disorder (MDD) is a mental health issue characterized by core features of “depressed mood” and “loss of interest or pleasure” and accompanied by symptoms such as fatigue, neurocognitive impairment, and sleep disturbances (Kennedy, 2008). Approximately 300 million of the global population experience MDD, and the prevalence rate is increasing (Liu et al., 2020; Monroe and Harkness, 2022). Despite the availability of various antidepressant medications, the rates of response are modest and patients may experience severe side effects or remission (Gorzalka and Hill, 2011). Therefore, overcoming the inadequate response to MDD treatment is a critical challenge. Concerns about the limited effectiveness of current depression treatment strategies have prompted a search for emerging drugs and therapies. In this context, traditional Chinese medicine (TCM), with its comparatively high translational value, may offer new possibilities for targeted therapy for MDD.

In recent years, the cultivation of Saposhnikoviae Radix (SR) in northeast China has regained momentum because of its high medicinal and dietary value (Yang et al., 2020b). A recent metabolomics study showed that the SR extract holds potential medicinal prospects in neuroinflammatory defense by inhibiting pro-inflammatory genes and upregulating metabolites such as levodopa-dopamine, gamma-hydroxybutyric acid, and 5-hydroxy-L-tryptophan (Zhu et al., 2021). As one of the most abundant components in SR root, 5-O-methylvisammioside (MeV) is a marker compound used to determine the genuineness and quality control of SR in the Chinese Pharmacopoeia (Xu et al., 2024b). Therefore, the medicinal value of MeV has significant pharmacological importance. MeV was shown to have antipyretic and analgesic functions (Yang et al., 2017), and to treat psoriasis-like dermatitis (Fu et al., 2020). Recently, MeV was demonstrated to exert an antidepressant effect by inhibiting the nuclear/cytosolic ratio of nuclear factor kappa beta (NF-κB) and upregulating the levels of brain-derived neurotrophic factor (BDNF) and tropomyosin receptor kinase B (TrkB) in the hippocampus of LPS mice (Sun et al., 2020). MeV also increased superoxide dismutase levels and decreased malondialdehyde content in the hippocampus. Following MeV treatment *in vitro*, the overactivation of BV-2 microglia was inhibited via the NF-κB/inhibitory subunit of NF kappa B alpha (IκBα) pathway (Sun et al., 2020). MeV may also play a neuroprotective role in focal cerebral ischemia in rats by regulating the phosphatidylinositol 3-kinase/protein kinase B signaling pathway in the cerebral cortex (Chang and Wu, 2016). No studies have explored the utility of MeV in treating high-risk mental disorders such as MDD.

With the convergence and popularization of bioinformatics and systems biology, network pharmacology has become a more convenient approach for deciphering the intrinsic mechanisms of drug therapy for diseases in the form of visual “network targets” (Gu et al., 2022). The intervention mechanisms of candidate compounds, targets, and diseases are interlinked through network pharmacology, which provides a statistical basis for systematically characterizing how drugs treat diseases (Li and Zhang, 2013). The multi-channel regulatory mechanism emphasized by network pharmacology is similar to the pharmacodynamic characteristics of multi-targets and multi-pathways in TCM (Zhang et al., 2023). Network pharmacology has significantly enhanced the elucidation of the multi-faceted targets of TCM and the anti-depression mechanisms (Zhang et al., 2021; Cao et al., 2023; Chen et al., 2024).

Research has demonstrated that MeV possesses excellent antioxidant and anti-inflammatory properties. Therefore, we hypothesized that MeV may play a potential antidepressant role in the hippocampus and microglia. Because of its low oral availability, MeV is often overlooked when screening for the effective ingredients of SR using drug similarity and oral bioavailability values in the TCM Systems Pharmacology Database and Analysis Platform. Therefore, in this study, MeV was used as an independent object for network pharmacological research, combined with molecular docking to calculate and analyze targets and pathways for treating depression. Lipopolysaccharide (LPS), an inflammatory inducer, has been widely used to create a depression model in mice that mimics the neuroinflammation-related symptoms observed in patients with mood or anxiety disorders (van Eeden et al., 2020; Xu et al., 2023). LPS triggers immune responses and neurochemical changes in the brain, including secretion of proinflammatory cytokines and activation of microglia (Yu et al., 2022).

Repeated exposure to LPS is used to establish a mouse model of depression characterized by neuroimmune disorders, leading to depression-like behaviors and an “inflammatory storm” (Li et al., 2021a; Xu et al., 2024a). The binding of Toll-like receptor 4 (TLR4), which is expressed on the membrane of immune cells to LPS, activates the NF-κB signaling pathway through proteasomal degradation of IκB and phosphorylation of NF-κB subunits p65 (Yu et al., 2020; Li et al., 2024). The hyperactivity of NF-κB induces the release of proinflammatory cytokines such as interleukin-1 beta (IL1β) and activates microglial cells, resulting in neural injuries observed in mood disorders (Lynall et al., 2020; Mello et al., 2021).

The proto-oncogene tyrosine-protein kinase SRC is a prototype member of the Src family of kinases and is highly expressed in various cell types involved in regulating secretion (Kumar et al., 2015). Research indicates that SRC in the brain is sensitive to stress and can undergo a series of adaptive changes throughout the duration of depression (Wang et al., 2022). SRC is activated through autophosphorylation in response to stress, potentially influencing susceptibility to depression. However, active SRC may also play a dynamic role in the molecular mechanisms underlying antidepressant therapy (Abe et al., 2019). In this study, we conducted biological experiments to validate the primary targets and pathways. We established a mouse model of depression through intraperitoneal injection of 1 mg/kg LPS over 3 days and used the model to explore the molecular mechanisms by which MeV improves biochemical distortions in the hippocampus. This work provides an important theoretical basis for exploring the antidepressant pathways affected by MeV and the regulatory targets for treating MDD.

## Methods

### Target collection

The Simplified Molecular Input Line Entry System and molecular formula of MeV were extracted from the PubChem public database (https://pubchem.ncbi.nlm.nih.gov/; Kim et al., 2016) and imported into the SwissTargetPrediction database (http://www.Swisstargetprediction.ch/; Daina et al., 2019) and the PharmMapper database (http://www.lilab-ecust.cn/pharmmapper/; Wang et al., 2017) to acquire potential targets.

To search for target genes and compile targets based on their scores, keywords related to “major depressive disorder” were entered into three public databases: DrugBank (https://www.drugbank.ca/; Wishart et al., 2018), GeneCards (https://www.genecards.org/; Stelzer et al., 2016), and DisGeNET (https://www.disgenet.org/; Piñero et al., 2020). The collected targets were standardized to gene symbols using the UniProt database (https://www.uniprot.org/; UniProt Consortium, 2023), and duplicates were removed.

### Network construction

Venny 2.1 (https://bioinfogp.cnb.csic.es/tools/venny/) was used to identify drug-disease overlapping genes as potential targets for MeV intervention in MDD. The common targets were imported into the Search Tool for the Retrieval of Interacting Genes (STRING) database (https://string-db.org; Szklarczyk et al., 2023) to analyze protein-protein interactions. Cytoscape 3.7 (https://cytoscape.org/; Doncheva et al., 2019) was used to visualize the network-target mapping of MeV-treated MDD and to calculate degree, betweenness centrality, and closeness centrality.

### Gene Ontology enrichment and Kyoto Encyclopedia of Genes and Genomes pathway analyses

Gene Ontology (GO) and Kyoto Encyclopedia of Genes and Genomes (KEGG) pathway enrichment analyses of the common targets were performed using the Metascape database (v3.5.20230101, http://www.metascape.org/; Zhou et al., 2019). GO analysis included three modules: biological processes (BP), molecular functions (MF), and cellular components (CC). The results were filtered using *P* < 0.01, a minimum count of 3, and an enrichment factor > 1.5.

### Molecular docking validation

The three-dimensional structures of MeV and Src were retrieved from the PubChem database and the Research Collaboratory for Structural Bioinformatics Protein Data Bank (RCSB PDB) database (https://www.rcsb.org/; Burley et al., 2023), respectively. PDBQT format files, processed and generated by AutoDockTools (MGLTools 1.5.7, https://ccsb.scripps.edu/mgltools/; Morris et al., 2008), were used for molecular docking. The docking conformation between MeV and Src with the lowest binding energy was selected and imported into PyMOL software (version 2.4.0, http://www.pymol.org) for visualization.

### Animals

To avoid the interference of estrogen on neuroinflammation and microglia physiology in depressed mice (Villa et al., 2016; Zhang et al., 2024), male mice were used for the experimental study. Male Institute of Cancer Research mice, 4–6 weeks old and weighing 20 ± 2 g, were purchased from Changchun YiSi Experimental Animal Technology Co., Ltd. (Changchun, Jilin, China; license No. SCXK (Ji) 2018-0001). Mice were housed individually in plastic cages and acclimated in a feeding facility for 3 days. The animals were maintained on a 12-hour light/dark cycle at 23–26°C with 40%–60% humidity throughout the study. The experimental protocol was approved by the Institutional Animal Care and Use Committee of Jilin University (approval No. 2022375). All experiments were designed and reported following the Animal Research: Reporting of *In Vivo* Experiments (ARRIVE) guidelines (Percie du Sert et al., 2020).

**Figure 1:**
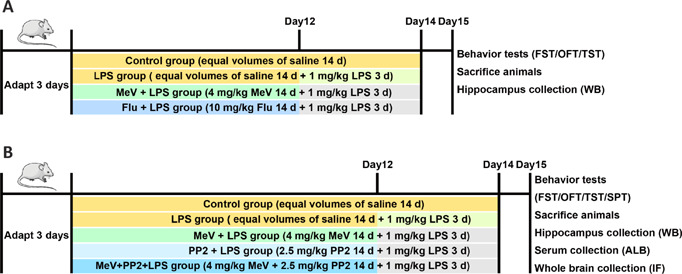
Experimental overview. (A) Mice were pretreated with MeV and Flu for 11 days before administration of LPS for 3 days to establish the LPS-induced depression model. Flu, an antidepressant, served as the positive control (*n* = 14). (B) In the second experiment, the SRC inhibitor PP2 was administered to the indicated groups by intraperitoneal injections for 14 days with LPS model establishment as described above. At the end of treatment, the mice underwent behavioral tests and were subsequently euthanized. Whole brain tissue, hippocampus, and serum were collected (*n* = 20). ALB: Albumin; Flu: fluoxetine; FST: forced swimming test; IF: immunofluorescence; LPS: lipopolysaccharide; MeV: 5-O-methylvisammioside; OFT: open field test; SPT: sucrose preference test; TST: tail suspension test; WB: Western blot.

In Experiment I (**Figure 1A**), we explored the effect of MeV on alleviating LPS-induced depression-like behaviors and associated signaling pathways, with or without MeV treatment for 14 days. Fluoxetine (Flu) was used as a positive control (Ren et al., 2015; Zhang et al., 2022). The mice were randomly divided into four groups (*n* = 14 per group; 7 mice were used for behavioral testing and 7 mice were used for the western blot assay): the control group, LPS group, LPS + MeV group, and LPS + Flu group. In Experiment II (**Figure 1B**), we investigated the role of SRC in the MeV treatment of LPS model mice by establishing two additional groups (*n* = 20 per group; 10 mice were used for behavioral testing, 6 mice were used for the western blot assay and 4 mice were used for immunofluorescence analysis): LPS + PP2 (an SRC inhibitor) group and LPS + PP2 + MeV group.

To establish the LPS-induced mouse model, mice were injected with LPS (1 mg/kg per day, days 12–14; Escherichia coli 055:B5, L2880-10mg, Sigma Aldrich, St. Louis, MO, USA). Control mice received equal amounts of saline. Saline, MeV (4 mg/kg per day; RS00021020, Nature-standard, Shanghai, China), Flu (10 mg/kg per day; PHR1394-1g, Sigma Aldrich), and PP2 (2.5 mg/kg per day; HY-13805, MedChemExpress, Monmouth Junction, NJ, USA) were administered via intraperitoneal injection using a 1 mL syringe on days 1–14. Injections were performed in the following order: PP2, MeV, and LPS, with a 60-minute interval between each injection.

Blood was collected by retro-orbital bleeding of mice after behavioral testing. The hippocampus was removed following anesthesia with pentobarbital sodium (50 mg/kg, intraperitoneal injection; Dingguo Changsheng Biotechnology, Beijing, China) and rapid decapitation. Mice were deeply anesthetized with a higher dose of pentobarbital sodium (60 mg/kg, intraperitoneal injection), and transcardiac perfusion was performed to extract brain tissue (Liu et al., 2023). The entire brain was fixed in 4% paraformaldehyde for immunofluorescence staining.

### Behavioral tests

Behavioral tests were performed 24 hours after the mice received the last dose of LPS. The procedures for behavioral testing were conducted following previously adapted protocols (Wang et al., 2019, 2021).

#### Open field test

The basin-shaped apparatus used for the open field test is shown in **Additional Figure 1**. The floor of the arena was divided into 16 square sections of essentially equal size. Mice were placed in the apparatus, and their movements were digitally recorded using a C930 webcam (Logitech International S.A., Newark, CA, USA) for 8 minutes. Horizontal movement was defined as the mouse crossing from one grid square to another, while vertical movement was defined as the mouse standing on its hind legs.

#### Tail suspension test

Mice were suspended by attaching the tip of their tails (1–2 cm from the end) to a beam using adhesive tape or a clip. Each test lasted for 8 minutes, and the total immobility time during the last 5 minutes was recorded. Mice were considered immobile when they ceased struggling and hung passively without limb movement.

#### Forced swimming test

Mice were tested for 7 minutes in a 25-cm high plastic cylinder filled with 12 cm of fresh water (25 ± 1°C). The total time of immobility, defined as the absence of movement except the slight limb movements necessary for the mice to keep their bodies afloat or balanced in the water, was evaluated during the last 5 minutes of the test.

#### Sucrose preference test

Mice were trained for sucrose consumption three times to establish sucrose preference prior to testing. On the third day of LPS administration, the mice were deprived of food and water for 12 hours. Bottles containing a 2% sucrose solution or tap water were placed in the cages, and the consumption of both the sucrose solution and tap water was recorded over a 12-hour period. Sucrose preference was calculated as follows: sucrose preference (%) = sucrose consumption/(sucrose consumption + water consumption) × 100.

### Western blot assay

The entire hippocampal tissue was homogenized in cold lysis buffer containing protease and phosphatase inhibitors (Epizyme Biotech, Shanghai, China). Protein lysates were separated on 10% or 12% sodium dodecyl sulfate-polyacrylamide gels and transferred onto polyvinylidene fluoride membranes with a pore size of 0.45 µm (Durapore® Millipore, Darmstadt, Germany) at 100 V for 1 hour. After blocking for 1 hour in 50 mL of 5% nonfat dry milk, the membranes were incubated overnight at 4°C with primary antibodies, followed by incubation with horseradish peroxidase-conjugated secondary antibodies for 2 hours at 25°C. Immunoreactive bands were visualized using enhanced chemiluminescence solution (Epizyme Biotech) in a gel imaging system (Tanon, Shanghai, China). The band intensity was quantified using ImageJ software (version 1.53, National Institutes of Health, Bethesda, MD, USA; Schneider et al., 2012). Total protein expression was normalized to β-actin, and phosphorylated protein expression was normalized to the corresponding total protein. The primary antibodies included antibodies against TLR4, NF-κB, phospho-NF-κB (p-NF-κB), IκBα, IL1β, Src (SRC), phospho-SRC (p-SRC), ionized calcium-binding adapter molecule 1 (IBA1), and β-actin; detailed information is listed in **Additional Table 1**.

**Additional Table 1:** Antibody information

Antibody	Species	Dilution	Supplier	Catalog number	RRID	Application
Anti-β-actin	Mouse	1:3000	TransGen Biotech (Beijing, China)	HC201-01	AB_2860007	Western blot assay
Anti-TLR4	Rabbit	1:1000	Immunoway (Suzhou, China)	YT0744	AB_3662106	Western blot assay
Anti-IBAl	Rabbit	1:1000	Immunoway	YN2165	AB_3662105	Western blot assay
Anti-IL1β	Rabbit	1:1000	Immunoway	YT2322	AB_2921215	Western blot assay
Anti-NF-κB p65	Rabbit	1:500	Beyotime (Shanghai, China)	AN365	AB_3662107	Western blot assay
Anti-phospho-NF-κB p65	Rabbit	1:500	Beyotime	AF5881	AB_3662108	Western blot assay
Anti-IκBα (1)	Rabbit	1:500	Bioss (Beijing, China)	bs-1287R	AB_10857466	Western blot assay
Anti-lKBa (2)	Rabbit	1:500	ABclonal (Wuhan, China)	A1187	AB_2758831	Western blot assay
Anti-SRC	Rabbit	1:1000	Cell Signaling Technology (Danvers, MA, USA)	#2109T	AB_2106059	Western blot assay
Anti-phospho-SRC	Rabbit	1:1000	Cell Signaling Technology	2101S	AB_331697	Western blot assay
HRP-conjugated anti-rabbit IgG	Goat	1:3000	ZSGB-Bio (Beijing, China)	ZB-2301	AB_2747412	Western blot assay
HRP-conjugated anti-mouse IgG	Goat	1:6000	ZSGB-Bio	ZB-2305	AB_2747415	Western blot assay
Anti-IBA1	Rabbit	1:1000	Abcam (Cambridge, UK)	ab178846	AB_2636859	Immunofluorescence staining
Anti-rabbit IgG (Alexa Fluor^®^ 488)	Goat	1:500	Abcam	ab150077	AB_2630356	Immunofluorescence staining

HRP: Horseradish peroxidase; IBA1: ionized calcium-binding adapter molecule 1; IκBα: inhibitory subunit of NF-κB alpha; IL1β: interleukin 1 beta; NF-κB: nuclear factor kappa-B; SRC: non-receptor tyrosine kinase Src; TLR4: Toll-like receptor 4.

### Serum albumin detection

Whole blood was allowed to clot for 60 minutes at 25°C (Fisher et al., 2017; Greenfield, 2017). The serum was separated and stored at –80°C. Serum albumin (ALB) was measured in the serum using the bromocresol green dye-binding method following the guidelines of the ALB assay kit (Nanjing Jiancheng Bioengineering Institute, Nanjing, China). ALB concentrations were measured at a wavelength of 628 nm using a multifunctional enzyme-labeling instrument (Thermo Scientific, Varioskan Flash, MA, USA).

### Immunofluorescence analysis

Stripped brain tissue was fixed overnight in 4% paraformaldehyde (Saint-Bio, Shanghai, China) and dehydrated in a gradient sucrose solution at 4°C. Twenty-micrometer sagittal plane brain sections were cut using a cryostat and stored at –20°C until the day of staining. After three rinses with phosphate-buffered saline, the sections were blocked with 5% serum for 1 hour to reduce non-specific staining. The sections were then incubated in anti-IBA1 antibody diluent at 4°C overnight. The sections were then incubated with an Alexa Fluor® 488-conjugated secondary antibody for 1 hour at 25°C on a gyrating platform. A microscope camera system (Cellsens, Tokyo, Japan) was used to obtain immunofluorescent images. The densitometry of IBA1-positive microglia was calculated using FIJI software (National Institutes of Health; Schindelin et al., 2012). Antibody information is listed in **Additional Table 1**.

### Statistical analysis

The sample size was mainly determined following previous studies that focused on screening antidepressant components in herbal medicine (Li et al., 2021b; Jiang et al., 2023). Data pertaining to accidental leakage of water or sucrose solution during the sucrose preference experiment and data from mice that died during the forced swim test were excluded from the analysis. Animal studies were conducted in a blinded manner, with the evaluator unaware of the group assignments for each mouse. Data were analyzed using IBM SPSS Statistics for Windows (version 25.0, IBM Corp., Armonk, NY, USA), and the results are presented as mean ± standard error of the mean (SEM). Parametric variables were statistically analyzed using one-way analysis of variance, followed by Tukey’s *post hoc* test, to assess differences between groups. A *P*-value < 0.05 indicated statistical significance.

## Results

### Network pharmacology analysis of the potential mechanism of 5-O-methylvisammioside in major depressive disorder

**Figure 2:**
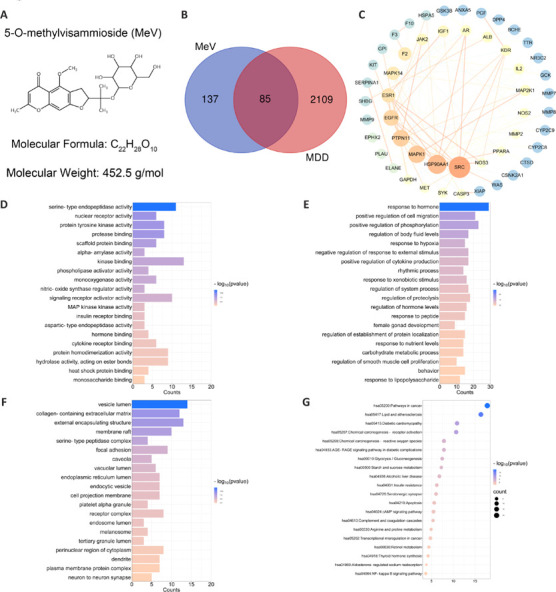
Network pharmacology analysis of MeV in MDD. (A) Structural formula of MeV. (B) Venn diagram of MeV and MDD targets. (C) The protein-protein interaction network of the 85 overlapping targets. Nodes represent proteins, with the color of each circle indicating the degree value. Edges between nodes represent protein–protein associations. (D–F) The top 20 items from GO functional enrichment analysis, including molecular function analysis (D), biological process analysis (E), and cellular component analysis (F). (G) KEGG enrichment analysis. GO: Gene Ontology; KEGG: Kyoto Encyclopedia of Genes and Genomes; MDD: major depressive disorder; MeV: 5-O-methylvisammioside.

The chemical structure of MeV is shown in **Figure 2A**. By conducting an integrated search across multiple databases, we identified 222 targets related to MeV and 2194 targets associated with MDD and extracted 85 overlapping targets (**Figure 2B**). Target information is provided in **Additional Table 2**. Protein-protein interaction networks for the overlapping targets were constructed using the STRING database, and the visualized network is presented in **Figure 2C**. The degree values of the targets are listed in **Additional Table 3**. These targets may represent important genes involved in the potential effects of MeV in MDD. The proto-oncogene tyrosine-protein kinase SRC showed the highest degree value and may be a core target of MeV treatment for MDD.

**Additional Table 2:** Common targets between MeV and MDD

Gene name	Protein name	Uniprot ID
*MMP9*	Matrix metalloproteinase-9	P14780
*PAH*	Phenylalanine-4-hydroxylase	P00439
*AMD1*	S-adenosylmethionine decarboxylase proenzyme	P17707
*AR*	Androgen receptor	P10275
*F3*	Tissue factor	P13726
*MAP2K1*	Dual specificity mitogen-activated protein kinase kinase 1	Q02750
*MIF*	Macrophage migration inhibitory factor	P14174
*ADH1B*	All-trans-retinol dehydrogenase	P00325
*CYP2C8*	Cytochrome P450 2C8	P10632
*NOS3*	Nitric oxide synthase	P29474
*ISG20*	Interferon-stimulated gene 20 kDa protein	Q96AZ6
*HPRT1*	Hypoxanthine-guanine phosphoribosyltransferase	P00492
*HSPA5*	Endoplasmic reticulum chaperone BiP	P11021
*SYK*	Tyrosine-protein kinase SYK	P43405
*IL2*	Interleukin-2	P60568
*ADORA1*	Adenosine receptor A1	P30542
*NCS1*	Neuronal calcium sensor 1	P62166
*TTR*	Transthyretin	P02766
*GPI*	Glucose-6-phosphate isomerase	P06744
*RORA*	Nuclear receptor ROR-alpha	P35398
*PPARA*	Peroxisome proliferator-activated receptor alpha	Q07869
*KDR*	Vascular endothelial growth factor receptor 2	P35968
*CASP3*	Caspase-3	P42574
*HSP90AA1*	Heat shock protein HSP 90-alpha	P07900
*ANXA5*	Annexin A5	P08758
*EPHX2*	Bifunctional epoxide hydrolase 2	P34913
*CTSD*	Cathepsin D	P07339
*ANG*	Angiogenin	P03950
*WAS*	Actin nucleation-promoting factor WAS	P42768
*TPH1*	Tryptophan 5-hydroxylase 1	P17752
*NR1H2*	Oxysterols receptor LXR-beta	P55055
*MAPK14*	Mitogen-activated protein kinase 14	Q16539
*ESRRG*	Estrogen-related receptor gamma	P62508
*SERPINA1*	Alpha-1-antitrypsin	P01009
*PPARG*	Peroxisome proliferator-activated receptor gamma	P37231
*ADORA2A*	Adenosine receptor A2a	P29274
*PGF*	Placenta growth factor	P49763
*KIF11*	Kinesin-like protein KIF11	P52732
*DPP4*	Dipeptidyl peptidase 4	P27487
*PTPN11*	Tyrosine-protein phosphatase non-receptor type 11	Q06124
*PLAU*	Urokinase-type plasminogen activator	P00749
*APRT*	Adenine phosphoribosyltransferase	P07741
*CSNK2A1*	Casein kinase II subunit alpha	P68400
*GAPDH*	Glyceraldehyde-3-phosphate dehydrogenase	P04406
*ALB*	Serum albumin	P02768
*REN*	Renin	P00797
*IMPA1*	Inositol monophosphatase 1	P29218
*PDE4B*	cAMP-specific 3',5'-cyclic phosphodiesterase 4B	Q07343
*SRC*	Proto-oncogene tyrosine-protein kinase Src	P12931
*F10*	Coagulation factor X	P00742
*EGFR*	Epidermal growth factor receptor	P00533
*TYMS*	Thymidylate synthase	P04818
*MAPK1*	Mitogen-activated protein kinase 1	P28482
*MMP8*	Neutrophil collagenase	P22894
*AMY1A*	Alpha-amylase 1A	P0DUB6
*AMY1C*	Alpha-amylase 1C	P0DTE8
*GSR*	Glutathione reductase, mitochondrial	P00390
*SDS*	L-serine dehydratase/L-threonine deaminase	P20132
*NR3C2*	Mineralocorticoid receptor	P08235
*SLC2A1*	Solute carrier family 2	P11166
*GSK3B*	Glycogen synthase kinase-3 beta	P49841
*PDE4D*	cAMP-specific 3',5'-cyclic phosphodiesterase 4D	Q08499
*AMY1B*	Alpha-amylase 1B	P0DTE7
*ESR1*	Estrogen receptor	P03372
*CTSK*	Cathepsin K	P43235
*CYP2C9*	Cytochrome P450 2C9	P11712
*ADH1C*	Alcohol dehydrogenase 1C	P00326
*BMP7*	Bone morphogenetic protein 7	P18075
*XIAP*	E3 ubiquitin-protein ligase XIAP	P98170
*F2*	Prothrombin	P00734
*JAK2*	Tyrosine-protein kinase JAK2	O60674
*KIT*	Mast/stem cell growth factor receptor Kit	P10721
*SELP*	P-selectin	P16109
*GCK*	Hexokinase-4	P35557
*BCHE*	Cholinesterase	P06276
*SHBG*	Sex hormone-binding globulin	P04278
*IGF1*	Insulin-like growth factor I	P05019
*ALDH2*	Aldehyde dehydrogenase, mitochondrial	P05091
*ELANE*	Neutrophil elastase	P08246
*B3GAT1*	Galactosylgalactosylxylosylprotein 3-beta-glucuronosyltransferase 1	Q9P2W7
*MET*	Hepatocyte growth factor receptor	P08581
*SOD2*	Superoxide dismutase	P04179
*NOS2*	Nitric oxide synthase, inducible	P35228
*MMP7*	Matrilysin	P09237
*MMP2*	72 kDa type IV collagenase	P08253

MDD: Major depressive disorder; MeV: 5-O-methylvisammioside.

**Additional Table 3:** Summary of target values including degree, betweenness centrality, and closeness centrality

Gene name	Degree	Betweenness centrality	Closeness centrality
*SRC*	19	0.274143	0.5
*HSP90AA1*	16	0.207146	0.44954128
*MAPK1*	14	0.152303	0.45794393
*PTPN11*	13	0.046707	0.40495868
*EGFR*	12	0.066518	0.41880342
*ESR1*	10	0.072167	0.40495868
*F2*	9	0.317032	0.42608696
*MAPK14*	9	0.043598	0.39837398
*IGF1*	7	0.026868	0.41525424
*JAK2*	7	0.023566	0.39516129
*ALB*	6	0.266752	0.33793103
*AR*	6	0.084434	0.41176471
*MAP2K1*	6	0.0127	0.39516129
*IL2*	6	0.004782	0.3740458
*KDR*	6	0.044312	0.38582677
*NOS2*	5	0.118545	0.38582677
*CASP3*	4	0.080782	0.34027778
*GAPDH*	4	0.079932	0.33793103
*MET*	4	0	0.37121212
*NOS3*	4	9.99E-04	0.33793103
*PPARA*	4	0.033637	0.40495868
*MMP2*	4	0.003594	0.36567164
*SYK*	4	6.98E-04	0.35766423
*APRT*	3	1	1
*EPHX2*	3	0.080782	0.28654971
*PLAU*	3	0.005585	0.37984496
*ELANE*	3	0.080782	0.21212121
*ADH1B*	2	0	1
*ALDH2*	2	0	1
*ADH1C*	2	0	1
*SERPINA1*	2	0.117347	0.26203209
*SHBG*	2	0.027249	0.33108108
*HSPA5*	2	0	0.32236842
*F10*	2	0	0.30246914
*F3*	2	0	0.30246914
*GPI*	2	0.040816	0.2565445
*KIT*	2	0	0.30434783
*MMP9*	2	0	0.33793103
*TTR*	1	0	0.25388601
*BCHE*	1	0	0.25388601
*DPP4*	1	0	0.25388601
*AMY1A*	1	0	1
*AMY1B*	1	0	1
*ANXA5*	1	0	0.25520833
*HPRT1*	1	0	0.6
*PDE4B*	1	0	0.6
*PDE4D*	1	0	0.6
*GSK3B*	1	0	0.29341317
*XIAP*	1	0	0.25520833
*CSNK2A1*	1	0	0.31210191
*CTSD*	1	0	0.28994083
*CYP2C8*	1	0	0.22374429
*CYP2C9*	1	0	0.22374429
*MMP8*	1	0	0.17562724
*MMP7*	1	0	0.17562724
*GCK*	1	0	0.20502092
*NR3C2*	1	0	0.31210191
*PGF*	1	0	0.28
*KIF11*	1	0	1
*TYMS*	1	0	1
*WAS*	1	0	0.33561644

**Figure 3:**
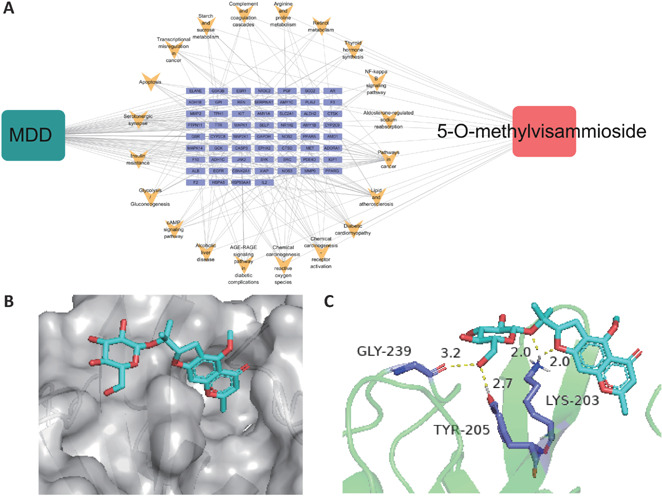
Prediction of the target-pathway network and molecular docking conformation of SRC and MeV. (A) The network illustrates the relationship between genes and pathways involved in the treatment of MDD with MeV. (B, C) Specific docking sites of MeV and SRC are shown, with the side chains of interacting amino acid residues between the ligands and target proteins displayed. Hydrogen bonds are represented as dashed lines. MDD: Major depressive disorder; MeV: 5-O-methylvisammioside; SRC: proto-oncogene tyrosine-protein kinase Src.

To further explore the possible mechanisms for MeV in MDD, GO and KEGG analyses of the potential targets were conducted using the Metascape database. GO enrichment analysis revealed 1113 items, including 102 MF, 947 BP, and 64 CC items. Using a filtering criterion of *P* < 0.01, we selected the top 20 significant enrichment terms from these three categories. The MF results shown in **Figure 2D** indicated that the targets were primarily enriched in kinase binding and serine-type endopeptidase activity. The BP results suggested that the potential targets of MeV for improving MDD focused mainly on responses to hormones, positive regulation of phosphorylation and cytokine production, and responses to lipopolysaccharide (**Figure 2E**). The enriched CC terms included vesicle lumen, dendrite, and neuron-to-neuron synapse (**Figure 2F**). A total of 147 KEGG enrichment signaling pathways were screened (with a selection criterion of *P* < 0.01), and the top 20 pathways are shown in **Figure 2G**. The NF-κB signaling pathway (hsa04064) and cAMP signaling pathway (hsa04024) are particularly notable pathways. Cytoscape software was used to generate the network relationships among the targets and pathways (**Figure 3A** and **Additional Table 4**). These findings suggest that MeV may exhibit therapeutic effects in MDD by interfering with inflammation-related processes and signaling pathways.

**Additional Table 4:** The top 20 terms enriched in KEGG analysis

Term	Description	Log P	Log (*q*-value)	Gene
hsa05200	Pathways in cancer	-17.8329071	-15.286	*XIAP/AR/CASP3/EGFR/ESR1/F2/GSK3B/HSP9fJAA1/IGF1/IL2/JAK2/KIT/MET/MMP2/MMP9/NOS2/PGF/ PPARG/MAPK1/MAP2K1/SLC2A1*
hsa05417	Lipid and atherosclerosis	-16.35984931	-14.114	*CASP3/MAPK14/CYP2C8/CYP2C9/GSK3B/HSPA5/HSP90AA1/JAK2/MMP9/NOS3/PPARG/MAPK1/SELP/SOD 2*/*SRC*
hsa05415	Diabetic cardiomyopathy	-10.87257625	-9.280	*MAPK14*/*CTSD*/*GAPDH*/*GSK3B*/*GSR*/*MMP2*/*MMP9*/*NOS3*/*PPARA*/*REN*/*SLC2A1*
hsa05207	Chemical carcinogenesis - receptor activation	-10.66899004	-9.168	*XIAP/AR/EGFR/EPHX2/ESR1/HSP90AA1/JAK2/PPARA/MAPK1/MAP2K1/SRC*
hsa05208	Chemical carcinogenesis - reactive oxygen species	-7.849944733	-6.625	*MAPK14/EGFR/EPHX2/MET/MAPK1/MAP2K1/PTPN11/SOD2/SRC*
hsa04933	AGE-RAGE signaling pathway in diabetic complications	-7.849243082	-6.625	*CASP3*/*MAPK14*/*F3*/*JAK2*/*MMP2*/*NOS3*/*MAPK1*
hsa00010	Glycolysis / Gluconeogenesis	-7.440476625	-6.274	*ADH1B*/*ADH1C*/*ALDH2*/*GAPDH*/*GCK*/*GPI*
hsa00500	Starch and sucrose metabolism	-7.256948605	-6.158	*AMY1A*/*AMY1B*/*AMY1C*/*GCK*/*GPI*
hsa04936	Alcoholic liver disease	-6.79647726	-5.755	*ADH1B*/*ADH1C*/*ALDH2*/*CASP3*/*MAPK14*/*GSK3B*/*PPARA*
hsa04931	Insulin resistance	-6.197687678	-5.253	*GSK3B*/*NOS3*/*PPARA*/*PTPN11*/*SLC2A1*/*NR1H2*
hsa04726	Serotonergic synapse	-6.037061833	-5.114	*CASP3*/*CYP2C8*/*CYP2C9*/*MAPK1*/*MAP2K1*/*TPH1*
hsa04210	Apoptosis	-5.61144349	-4.789	*XIAP*/*CASP3*/*CTSD*/*CTSK*/*MAPK1*/*MAP2K1*
hsa04024	cAMP signaling pathway	-5.454423264	-4.656	*ADORA1*/*ADORA2A*/*PDE4B*/*PDE4D*/*PPARA*/*MAPK1*/*MAP2K1*
hsa04610	Complement and coagulation cascades	-5.339302504	-4.571	*F2/F3/F10/SERPINA1/PLAU*
hsa00330	Arginine and proline metabolism	-4.912903947	-4.173	*ALDH2*/*AMD1*/*NOS2*/*NOS3*
hsa05202	Transcriptional misregulation in cancer	-4.740289089	-4.033	*ELANE*/*IGF1*/*MET*/*MMP9*/*PLAU*/*PPARG*
hsa00830	Retinol metabolism	-4.381113734	-3.721	*ADH1B*/*ADH1C*/*CYP2C8*/*CYP2C9*
hsa04918	Thyroid hormone synthesis	-4.213713252	-3.589	*ALB*/*GSR*/*HSPA5*/*TTR*
hsa04960	Aldosterone-regulated sodium reabsorption	-3.806070074	-3.255	*IGF1*/*NR3C2*/*MAPK1*
hsa04064	NF-kappa B signaling pathway	-3.662794145	-3.137	*XIAP/CSNK2A1/PLA U/SYK*

KEGG: Kyoto Encyclopedia of Genes and Genomes.

Molecular docking is a computer-aided technique for predicting binding sites and binding affinities of ligand-receptor interactions (Pinzi and Rastelli, 2019). This technology is often combined with network pharmacology to study the binding characteristics of small compounds or macromolecules to receptors, providing a new logical pathway for discovering the pharmacological properties of potential drugs (Luo et al., 2023; Pei et al., 2024). Crystal structures were selected for the docking study, with the primary focus on high-resolution structures that were experimentally determined mainly through X-ray crystallography (Vakser, 2014). High-resolution docking explores a finer conformational space in terms of resolution (Å) compared with low-resolution docking, providing more detailed predictions (Park et al., 2015). From the results of the network pharmacological analysis, SRC (RCSB PDB ID: 1A07; method: X-ray diffraction; resolution: 2.20 Å) was chosen for molecular docking. The binding poses and interactions between MeV and SRC were investigated using the AutoDock 4 program and its algorithm. The amino acid interactions between MeV and SRC are illustrated in **Figures 3B** and **C**. MeV forms hydrogen bonds with GLY239, TYR205, and LYS203 in SRC. The docking binding energy of MeV with SRC is -5.907 kcal/mol, indicating a stable binding. This result suggests that MeV and SRC exhibited docking poses with a stable binding affinity in terms of binding energy.

### 5-O-methylvisammioside improves lipopolysaccharide-induced depressive-like behaviors in mice

**Figure 4:**
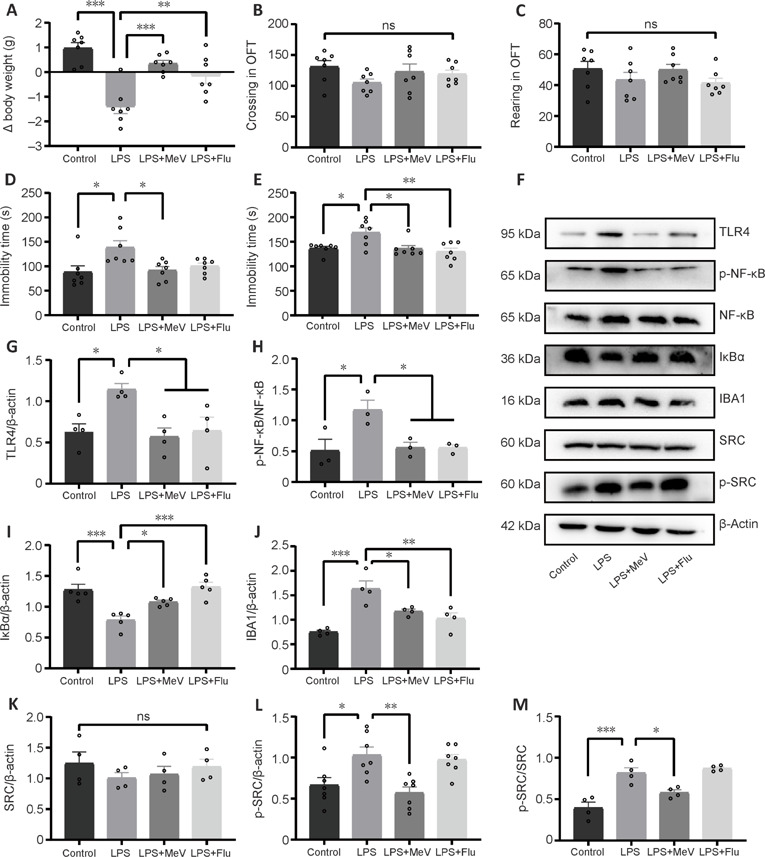
Effects of MeV on depression-like behaviors and hippocampal NF-κB pathway protein expression in mice with LPS-induced depression. (A) Effect of MeV and LPS on body weight (*n* = 7). (B, C) Results from the open field test (OFT) (*n* = 7). (D) Results from the tail suspension test (*n* = 7). (E) Results from the forced swimming test (*n* = 7). (F–M) Representative blots of TLR4, p-NF-κB, NF-κB, IκBα, IBA1, SRC, and p-SRC and bar graphs showing relative protein expression in mice treated with LPS, MeV, or Flu (*n* = 3–7). Data are presented as mean ± SEM. *P < 0.05, **P < 0.01, ***P < 0.001 (one-way analysis of variance followed by Tukey’s *post hoc* test). Flu: Fluoxetine; IBA1: ionized calcium-binding adapter molecule 1; IκBα: inhibitory subunit of NF-κB alpha; IL1β: interleukin 1 beta; LPS: lipopolysaccharide; MeV: 5-O-methylvisammioside; NF-κB: nuclear factor kappa B; ns: not significant; p-NF-κB: phospho-NF-κB; SRC: non-receptor tyrosine kinase Src; p-SRC: phospho-SRC; TLR4: toll-like receptor 4.

To verify the findings of network pharmacology, we established a mouse model of depression using LPS. We selected a dosage of 4 mg/kg MeV following a previous study (Sun et al., 2020). Flu, an antidepressant drug, was used as a positive control. Weight gain was significantly reduced in the LPS model mice compared with control mice, and treatment with MeV or Flu prevented weight loss in mice exposed to LPS (*P* < 0.001; **Figure 4A**). While LPS treatment did not affect locomotor activity (**Figure 4B**) or rearing (**Figure 4C**), it significantly increased immobility time during the tail suspension test (*P* = 0.013; **Figure 4D**) and the forced swimming test (*P* = 0.019; **Figure 4E**). Notably, treatment with MeV reduced immobility time and was similar to Flu levels. There was no significant change in the performance of MeV-treated mice compared to the Flu groups in both the tail suspension test and the forced swimming test. These results indicate that MeV significantly alleviated the depression-like behaviors induced by LPS.

### 5-O-methylvisammioside inhibits the lipopolysaccharide-induced activation of the nuclear factor kappa B pathway in the hippocampus

We next examined the effects of MeV on the NF-κB signaling pathway in the hippocampus of mice from the experimental groups (**Figure 4F–M**). LPS may activate NF-κB and its related pathway proteins through TLR4, leading to neuroinflammation, which plays a crucial role in the pathogenesis of depression (Jiang et al., 2023). Unsurprisingly, we found that LPS treatment upregulated the expression of TLR4 (*P* = 0.033) and IBA1 (*P* < 0.001) and decreased the expression of IκBα (*P* = 0.001). LPS also significantly increased the p-NF-κB/NF-κB ratio (*P* = 0.031). Both MeV and Flu reversed these changes in NF-κB pathway proteins induced by LPS treatment. We also observed increases in p-SRC (*P* = 0.020) and the p-SRC/SRC ratio (*P* < 0.001) in LPS-treated mice, and notably these changes were reduced by MeV but not by Flu. These data suggest that MeV may exert an antidepressant effect by inhibiting the activation of the NF-κB pathway in the hippocampus. MeV also reversed LPS-induced changes in p-SRC levels in the hippocampus and may induce antidepressant effects by an SRC-related mechanism.

### The SRC inhibitor PP2 reverses the activation of the nuclear factor kappa B pathway

Next, we investigated effects of SRC in LPS model mice using the SRC inhibitor PP2. Because of the limited data available on PP2 inhibitors in depression studies, we referenced the dosages used in mouse models of Parkinson’s disease. Previous research showed that 2.5 mg/kg PP2 positively influenced motor behavior and neuroinflammation in the Parkinson’s disease mouse model without causing adverse effects (Yang et al., 2020a). Therefore, we used 2.5 mg/kg PP2 in this study.

**Figure 5:**
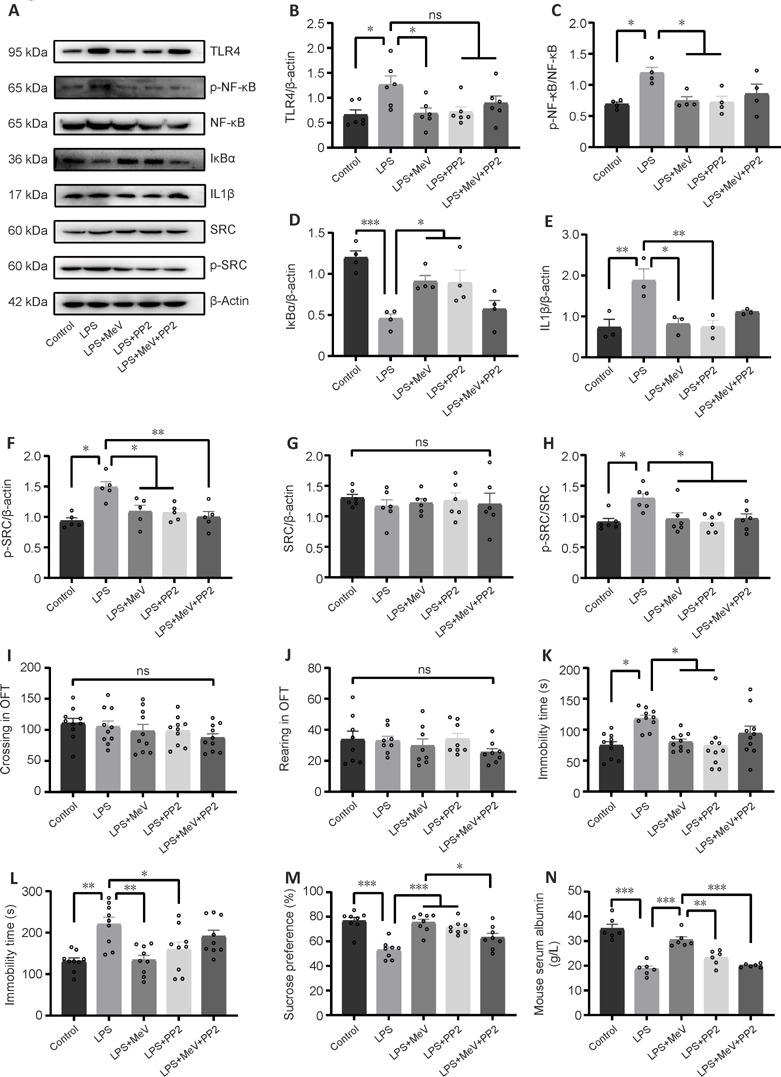
Effects of SRC inhibition on NF-κB pathway protein expression and depression-like behaviors in mice with LPS-treated. (A–H) Representative blots of TLR4, p-NF-κB, NF-κB, IκBα, IL1β, p-SRC, and SRC and quantification of protein expressions (*n* = 3–6). (I, J) Results from the open field test (OFT) (*n* = 10). (K) Results from the tail suspension test (*n* = 10). (L) Results from the forced swimming test (*n* = 9). (M) Results from the sucrose preference test (*n* = 8). (N) Serum ALB levels in mice (*n* = 6). Data are presented as mean ± SEM. *P < 0.05, **P < 0.01, ***P < 0.001 (one-way analysis of variance followed by Tukey’s *post hoc* test). ALB: Albumin; IBA1: ionized calcium-binding adapter molecule 1; IκBα: inhibitory subunit of NF-κB alpha; IL1β: interleukin 1 beta; LPS: lipopolysaccharide; NF-κB: nuclear factor kappa B; ns: not significant; p-NF-κB: phospho-NF-κB; PP2: SRC inhibitor; SRC: non-receptor tyrosine kinase Src; p-SRC: phospho-SRC; TLR4: Toll-like receptor 4.

We first examined the effects of PP2 on the NF-κB signaling pathway. SRC serves as a key regulatory factor that maintains numerous cellular processes, including cell proliferation and migration, and is involved in signal transduction related to inflammation (Cai et al., 2021). SRC is the upstream protein of the NF-κB pathway, and inhibition of SRC autophosphorylation leads to decreased activation of the downstream proteins of SRC (Kim et al., 2020). The activity of SRC is tightly controlled by phosphorylation at specific sites, with phosphorylation at tyrosine 416 recognized as a marker of SRC activation (Wu et al., 2015). Western blot analysis (**Figure 5A–H**) of the hippocampus of mice from the experimental groups showed that PP2 did not markedly alter TLR4 protein levels but it inhibited the LPS-induced upregulation of IL1β (*P* = 0.009) and p-SRC (*P* = 0.013) expression and the changes in the p-NF-κB/NF-κB (*P* = 0.049) and p-SRC/SRC (*P* = 0.010) ratios compared with LPS. PP2 also significantly increased the protein levels of IκBα (*P* = 0.014), similar to the effects of MeV. Furthermore, compared with the other four experimental groups, the combination therapy of MeV and PP2 did not markedly alter the expression of TLR4, IκBα, IL1β proteins, and the p-NF-κB/NF-κB ratio. Co-administration of MeV and PP2 significantly reversed the LPS-induced changes in p-SRC levels (*P* = 0.003) and the p-SRC/SRC ratio (*P* = 0.036). These results further indicate that the antidepressant effect of MeV may depend on its regulation of p-SRC to effectively suppress the activation of the NF-κB pathway.

### Inhibition of SRC alleviates lipopolysaccharide-induced depression-like behaviors in mice

We then examined the effects of MeV and PP2 treatment on the behavior of LPS mice, after confirming that 2.5 mg/kg PP2 effectively inhibited LPS-induced SRC activation. There were no significant changes in locomotor activity or rearing among the groups of mice in the open field test within the first 5 minutes (**Figure 5I** and **J**). Similar to the effects of MeV, PP2 administration reduced immobility time in the tail suspension test (*P* = 0.011) and forced swimming test (*P* = 0.035) and increased sucrose preference (*P* < 0.001) compared with LPS treatment (**Figure 5K–M**). No marked differences were observed between MeV and PP2 groups in the tail suspension test, forced swimming test, and sucrose preference test. These results suggest that PP2 alleviated depression-like behaviors in LPS-treated mice and exhibited effects similar to those observed in MeV-treated mice. We also observed no significant change in the performance of mice treated with a combination of MeV and PP2 compared to the other groups (including LPS, MeV, and PP2 administration) in both the tail suspension test and the forced swimming test. However, compared to the MeV treatment group, the sucrose preference of mice treated with MeV combined with PP2 was significantly reduced (*P* = 0.023). This finding suggests that the administration of PP2 in LPS-treated mice may attenuate the effects of MeV. TCM suggests that SR and its components alleviate depression through the spleen-liver meridian channel (Liang et al., 2017; Liu et al., 2022). ALB, primarily produced in the liver, is the most abundant protein in the blood and plays a crucial role in transporting metabolites and maintaining nutritional balance (Shojai et al., 2022; Ullah et al., 2023).

Our data showed that serum ALB levels were reduced in mice exposed to LPS, and MeV treatment restored ALB levels to near-normal values (*P* < 0.001; **Figure 5N**). However, neither PP2 nor the combination of MeV and PP2 affected ALB levels compared with LPS treatment. Compared with the MeV treatment group, the ALB levels of mice in the PP2 (*P* = 0.003) or PP2 combined with MeV treatment (*P* < 0.001) group were significantly downregulated. This suggests that while MeV counteracted the reduction of ALB induced by LPS, SRC does not appear to be involved in this process.

### 5-O-methylvisammioside or PP2 decreases IBA1 expression in the hippocampal CA1 and CA3 regions

**Figure 6:**
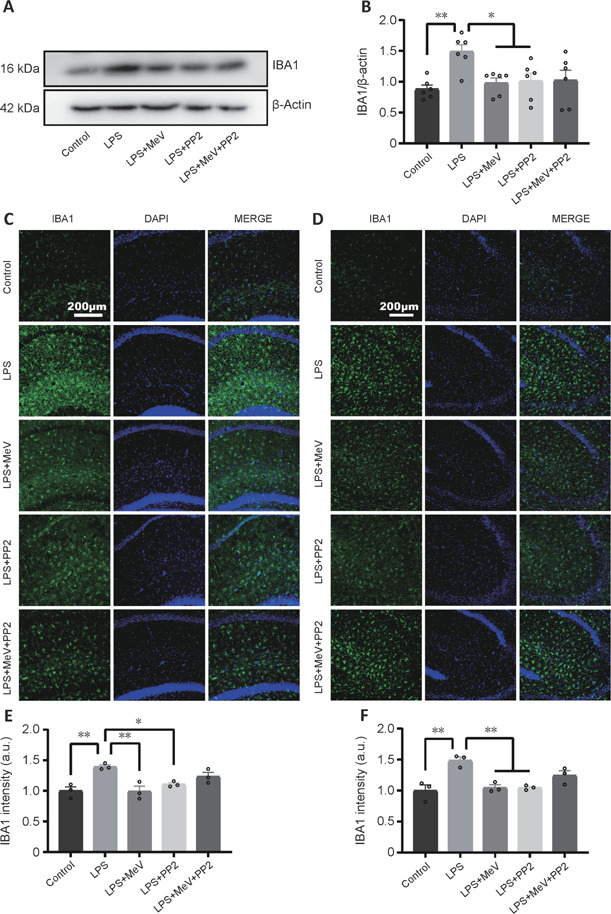
Effects of MeV treatment on microglial activation in mice with LPS-induced depression. (A, B) Representative western blot images showing IBA1 expression and quantification of protein expression (*n* = 6). (C, D) Representative fluorescence images of IBA1 (green) in the hippocampal CA1 and CA3 regions and quantification of relative intensity (*n* = 3). Scale bars: 200 µm. Data are presented as mean ± SEM. *P < 0.05, **P < 0.01 (one-way analysis of variance followed by Tukey’s *post hoc* test). CA: Cornu ammonis; IBA1: ionized calcium-binding adapter molecule 1; LPS: lipopolysaccharide; MeV: 5-O-methylvisammioside; PP2: SRC inhibitor.

Western blot and immunofluorescence analyses were conducted to examine the effects of MeV and the SRC inhibitor PP2 on IBA1 levels in the hippocampus. Studies found that excessive activation of microglia is involved in the pathogenesis of depression (Han et al., 2022; Yang et al., 2024). IBA1, an intracellular Ca^2+^-binding adapter protein, is often used as a marker for microglia activation (Lituma et al., 2021). As shown in **Figure 6A** and **B**, the LPS-induced increase in hippocampal IBA1 expression was reversed by treatment with MeV (*P* = 0.031) or PP2 (*P* = 0.049). Co-treatment of MeV with PP2 did not markedly reduce IBA1 levels in the hippocampus compared with LPS. Compared with the MeV group, there was no marked change in the expression of IBA1 in the MeV and PP2 group. Immunofluorescence also showed that LPS increased IBA1 expression in the hippocampal CA1 and CA3 regions compared with levels in the control group, while both MeV and PP2 reversed this trend (*P* < 0.05; **Figure 6C–F**). MeV combined with PP2 did not markedly decrease LPS-induced upregulation of IBA1 levels in the hippocampal CA1 and CA3 regions. There was no difference in the fluorescence intensity of IBA1 in the CA1 and CA3 regions between the MeV group and the group treated with MeV combined with PP2. These results suggest that SRC is likely involved in the regulatory effects of MeV on hippocampal microglia.

## Discussion

This study investigated the antidepressant effects and molecular mechanism of MeV from the drug-target-disease perspective using network pharmacology. Network pharmacological analysis indicated that MeV treatment for MDD is strongly associated with the NF-κB signaling pathway and the target SRC. Behavioral assessments and molecular biological results in the LPS-induced mouse model of depression showed that MeV/PP2 may alleviate LPS-induced depression-like behavior by regulating the expression of phosphorylated SRC and NF-κB pathway proteins. The combined treatment of MeV and PP2 in LPS mice resulted in a weakened antidepressive efficacy. Therefore, the antidepressant potential of MeV may partly stem from the inhibition of SRC-mediated activation of the NF-κB pathway.

The mechanism of MeV intervention in MDD was inferred by calculating and analyzing the interactive targets through network pharmacology. In addition to its effects on depression associated with medical conditions such as thyroid hormone imbalances (Baksi and Pradhan, 2021), diabetes (van der Feltz-Cornelis et al., 2021), alcoholic liver disease (Shaheen et al., 2021), vascular diseases (Tonhajzerova et al., 2020), and the pessimism associated with cancer in patients (Bortolato et al., 2017), MeV may also influence MDD by regulating factors related to kinase binding, inflammation, and cytokine production. Among these enriched pathways, the NF-κB signaling pathway has been widely recognized as one of the key pathways through which natural ingredients (such as Honokiol, Gypenoside-14, bergapten and raspberry ketone) can combat depression (Zhang et al., 2019; Jiang et al., 2023; Yan et al., 2023; Liu et al., 2024). Therefore, the NF-κB signaling pathway was identified as a critical pathway for MeV treatment of MDD, for further analysis. From the protein-protein interaction network results, we hypothesized that SRC is the primary target of MeV for the prevention and treatment of MDD. Molecular docking results demonstrated that MeV and SRC were well docked, suggesting that SRC, identified through network pharmacology, may be the target for MeV in the treatment of depression.

TLR4 binding LPS not only activates the NF-κB pathway but also induces SRC kinase responses through an increase in intracellular calcium ion concentration, thereby regulating cellular processes such as proliferation, survival, and migration (Thuringer et al., 2011; Bedini et al., 2017). SRC regulates the homeostatic functions of neurons and microglia, including membrane trafficking, gene expression, and the release of bioactive molecules (Portugal et al., 2022). Consequently, it is valuable to explore the relationship between SRC and the NF-κB signaling pathway in the context of the intervention of MeV in depression via a branched anti-inflammatory pathway. A preclinical study indicated that the persistence or exacerbation of MDD symptoms from neuroinflammation is associated with alterations in hippocampal function and microstructure (Belleau et al., 2019). In line with these findings, another clinical study reported enhanced neuroinflammation in the hippocampus of patients with depression, which may correlate with MDD recurrence and poor treatment responses (Dunjic-Kostic et al., 2013). Research has shown that LPS exposure significantly elevates cytokine levels and enhances NF-κB phosphorylation in the hippocampus, in addition to activating microglia, indicating that reducing hippocampal inflammation is essential for treating LPS-induced depression (Ali et al., 2020). Therefore, therapeutic strategies targeting hippocampal inflammation could potentially yield beneficial effects on the remission of depression, either directly or indirectly.

In this study, we successfully established the LPS-induced depression mouse model, as evidenced by significant weight loss, increased immobility time in tail suspension and forced swimming tests, and reduced sucrose consumption. Western blot experiments showed that LPS activated the NF-κB signaling pathway, leading to hippocampal inflammation. This was characterized by a reduction in IκBα protein expression, an increase in TLR4, IL1β, and IBA1 protein levels, and elevated p-NF-κB and p-SRC levels. LPS also stimulated microglial activation in the CA1 and CA3 regions of the hippocampus, as indicated by enhanced IBA1 immunostaining. These findings suggest an association between the NF-κB signaling pathway and the occurrence and progression of depression, consistent with previous studies (Domingues et al., 2018; Ali et al., 2020). Notably, administration of MeV for 14 days significantly alleviated the aforementioned depression-like behaviors in LPS-treated mice and reversed the changes in the expression of related proteins.

We examined whether SRC serves as a pivotal target for MeV in inhibiting NF-κB pathway activation in the LPS model mice. A previous study demonstrated that the methanol extract of Archidendron clypearia exhibits potent anti-inflammatory effects by suppressing NF-κB activation and its upstream signaling through the phosphorylation of SRC (Seok Yang et al., 2012). Inhibition of SRC by PP2 reduced the expression of inflammatory mediators in LPS-stimulated macrophages, further suggesting the significance of SRC in the inflammatory response. In the current study, results from behavioral tests, western blot analyses, and immunofluorescence staining indicated that the SRC inhibitor PP2 exerted effects comparable to those of MeV by alleviating depression-like behavior in mice and inhibiting activation of the SRC-mediated NF-κB signaling pathway and hippocampal microglia. We also evaluated the effects of MeV on LPS-induced depression-like behavior and NF-κB pathway protein expression following SRC inhibition by PP2. In mice treated with both MeV and PP2, the regulatory effect of MeV on NF-κB pathway proteins, depression-like behavior, and microglia was diminished, with the exception of the downregulation of p-SRC. This phenomenon suggests that the combined effects do not represent the sum of the individual effects of MeV and PP2, and instead, the inhibitory relationship between the two agents may influence the downstream effects of the combination treatment. This hypothesis cannot be confirmed by the existing research data, so further research is needed to verify it. Moreover, decreased ALB levels are considered a marker indicating activation of the inflammatory response system in patients with MDD (Maes et al., 1999). Our data showed that serum ALB levels were reduced in mice exposed to LPS, and MeV treatment restored ALB levels to near-normal values. This finding, combined with the antidepressant evaluation data mentioned above, suggests that the pharmacological effects of MeV on depression are consistent with its meridian theory in TCM applications. These findings strongly suggest that MeV may be a potential candidate herbal ingredient for treating diseases associated with dysregulation of the LPS-induced NF-κB pathway.

Our data suggests that MeV is a potential antidepressant. However, the multifaceted exploration of MeV will be needed before it becomes a clinically reliable antidepression. For example, it will be necessary to investigate the influence of different routes of administration (e.g., oral/injection/transdermal) on antidepression efficacy, and whether modification of the functional group of MeV by prodrug technology can effectively improve its oral bioavailability (Tijani et al., 2021). In the future, the evaluation system of TCM preparations based on the Delphi method and the superiority chart may promote the priority transformation of MeV in clinical settings on the basis of the above research results (Tang et al., 2024). Further research into MeV as a promising TCM ingredient for the treatment of MDD is warranted.

This study has several limitations. First, while drug and disease gene data were accessible from multiple public databases for network pharmacological analysis, it was difficult to ascertain the exact mechanism by which MeV may treat MDD because of the limited number of identified targets. Second, while we verified the effects of MeV in the hippocampus, other brain regions in LPS-treated mice are inevitably affected by neuroinflammation, making it challenging to completely rule out the influence of other emotion-regulating brain regions. Third, we inhibited SRC solely with PP2 and did not use gene knockout techniques. While PP2 inhibits SRC phosphorylation, its specificity is not comparable to that of gene knockout. These issues should be addressed in future studies.

In conclusion, we hypothesized the mechanism of MeV’s intervention in MDD through network pharmacology, and our subsequent biological experiments confirmed that the anti-inflammatory properties of MeV are crucial for alleviating LPS-induced depressive symptoms. The mechanism of MeV may be closely associated with the SRC-mediated activation of the NF-κB pathway and the polarization of microglia. Previous studies have demonstrated that MeV possesses anti-inflammatory, antioxidant, and antidepressant pharmacological activities. The current work provides new evidence that MeV may have significant anti-neuroinflammatory and antidepressant properties as a potential treatment for MDD and related disorders.

## Additional files:

***Additional Table 1:*** *Antibody information.*

***Additional Table 2:*** *Common targets between MeV and MDD.*

***Additional Table 3:*** *Summary of target values including degree, betweenness centrality, and closeness centrality.*

***Additional Table 4:*** *The top 20 terms enriched in KEGG analysis.*

***Additional Figure 1:*** *Schematic diagram of the open field test.*

Additional Figure 1 Schematic diagram of the open field test.
The basin-shaped arena was divided into 16 square sections of approximately equal size by three concentric circles and 10 lines. For the experiment, mice were placed at the center of the arena, and their line crossings and rearing behavior were monitored.

## Data Availability

*All relevant data are within the paper and its Additional files.*
